# Supplementary material for: A possible four-phase coexistence in a single-component system
Source: Nat Commun. 2016 Aug 25;7:12599. doi: 10.1038/ncomms12599 (PMC5007327; doi:10.1038/ncomms12599)
Supplement: Supplementary Information — Supplementary Figures 1-3 and Supplementary Notes 1-2 [file ncomms12599-s1.pdf]

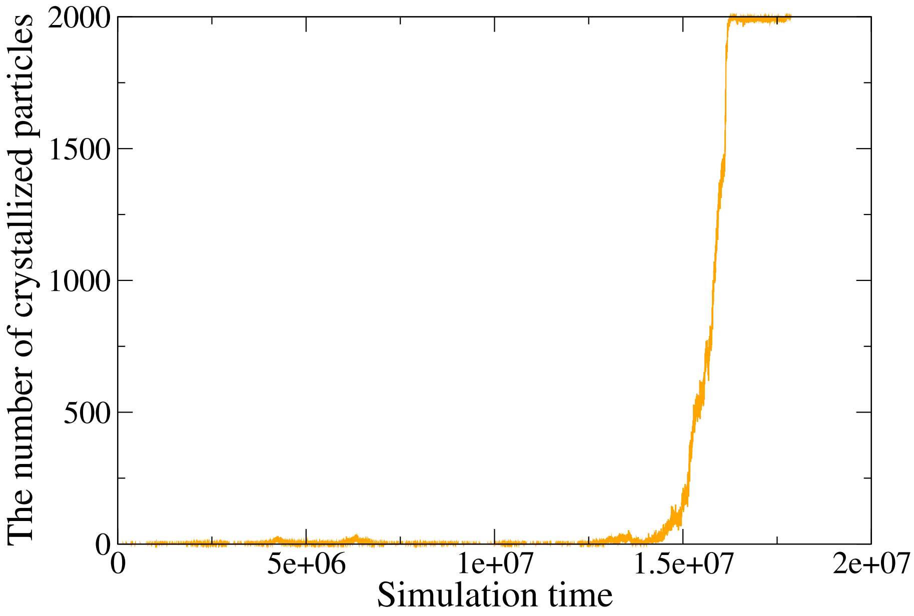

Supplementary Figure 1. **The number of particles crystallized to sc16 as a function of time.** The total number of particles in the simulation box is 2000.

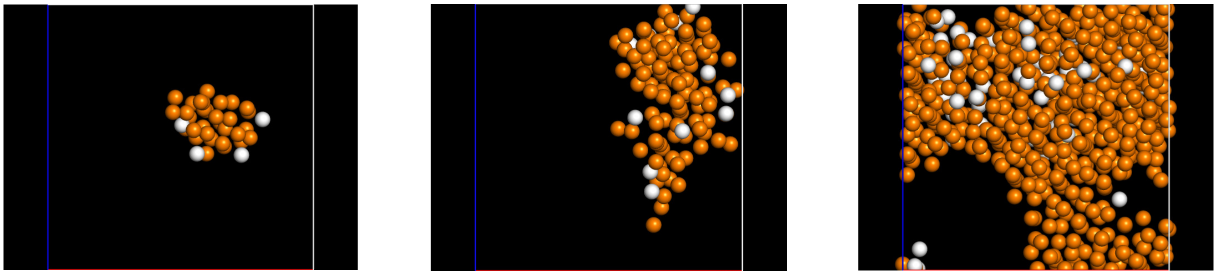

Supplementary Figure 2. **The configurations of particles crystallized to sc16 during crystallization.** Orange particles have the symmetry of sc16, whereas white particles represent defects.

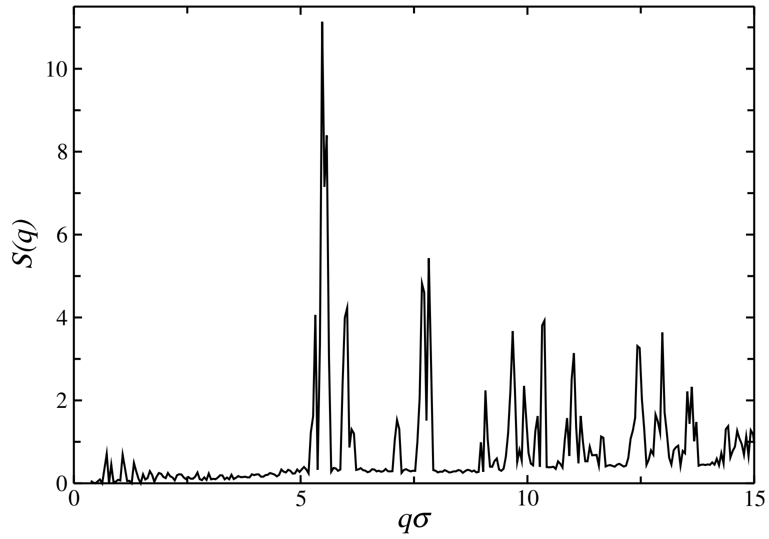

Supplementary Figure 3. **Structure factor of the X phase.** Static structure factor of the X phase.

SUPPLEMENTARY NOTE 1: DIRECT NUCLEATION OF THE SC16 PHASE

In order to confirm that fluid phase directly crystallizes in the sc16 phase, we perform Monte Carlo simulations in isothermal-isobaric ensembles at $\lambda = 21$, $P = 0.5$, and $T = 0.0395$. We use a liquid phase as initial configurations and run 50 independent trajectories, which all crystallize. Supplementary Figure 1 shows the number of particles crystallized to sc16 for one of these trajectories, and Supplementary Figure 2 shows the configurations of particles crystallized to sc16.

SUPPLEMENTARY NOTE 2: THE X PHASE

As discussed in the main manuscript, we have found a new stable crystal into which the β-tin phase transforms at low-temperatures. We have confirmed that this phase is not one of the other phases considered in this work. In Supplementary Figure 3 we plot the structure factor obtained from equilibrated configurations of the crystal. We leave for future work the classification of this crystalline phase.
